# Modulation of trigeminal neuropathic pain by optogenetic inhibition of posterior hypothalamus in CCI-ION rat

**DOI:** 10.1038/s41598-023-27610-7

**Published:** 2023-01-10

**Authors:** Jaisan Islam, Elina KC, Kyoung Ha So, Soochong Kim, Hyong Kyu Kim, Yoon Young Park, Young Seok Park

**Affiliations:** 1 Department of Medical Neuroscience, College of Medicine, Chungbuk National University, Cheongju, Republic of Korea; 2 Institute for Stem Cell and Regenerative Medicine (ISCRM), College of Veterinary Medicine, Chungbuk National University, Cheongju, Republic of Korea; 3 Bio-Max/N-Bio Institute, Institute of Bio-Engineering, Seoul National University, Seoul, Republic of Korea; 4 Department of Veterinary Medicine, College of Veterinary Medicine, Chungbuk National University, Cheongju, Republic of Korea; 5 Department of Medicine and Microbiology, College of Medicine, Chungbuk National University, Cheongju, Republic of Korea; 6 Department of Neurosurgery, Chungbuk National University Hospital, Cheongju, Republic of Korea; 7 Department of Neurosurgery, Chungbuk National University Hospital, College of Medicine, Chungbuk National University, 776, 1 Sunhwanro, Seowon-gu, Cheongju-Si, Chungbuk 28644 Republic of Korea

**Keywords:** Biological techniques, Biotechnology, Neuroscience

## Abstract

Posterior hypothalamus (PH), an important part of the descending pain processing pathway, has been found to be activated in trigeminal autonomic cephalalgias. However, there are very few studies conducted and information regarding its implications in trigeminal neuropathic pain (TNP). Therefore, we aimed to ascertain whether optogenetic inhibition of PH could affect the outcomes of a chronic constriction injury in the infraorbital nerve (CCI-ION) rat model. Animals were divided into the TNP animal, sham, and naive-control groups. CCI-ION surgery was performed to mimic TNP symptoms, and the optogenetic or null virus was injected into the ipsilateral PH. In vivo single-unit extracellular recordings were obtained from both the ipsilateral ventrolateral periaqueductal gray (vlPAG) and contralateral ventral posteromedial (VPM) thalamus in stimulation “OFF” and “ON” conditions. Alterations in behavioral responses during the stimulation-OFF and stimulation-ON states were examined. We observed that optogenetic inhibition of the PH considerably improved behavioral responses in TNP animals. We found increased and decreased firing activity in the vlPAG and VPM thalamus, respectively, during optogenetic inhibition of the PH. Inhibiting PH attenuates trigeminal pain signal transmission by modulating the vlPAG and trigeminal nucleus caudalis, thereby providing evidence of the therapeutic potential of PH in TNP management.

## Introduction

The posterior hypothalamus (PH), a periventricular region, is located in the diencephalon. The PH possesses reciprocal connections with areas involved in trigeminovascular and spinal pain processing that allows it to participate in several crucial physiological functions such as descending pain processing, circadian rhythm, feeding, thirst, and arousal^1^. Nevertheless, its involvement in cranial cephalalgias has attracted research interest, and many studies have been conducted to understand the underlying relationship between PH and headaches. Activation of the PH is a key feature of headaches and orofacial pain, which indicates primary dysfunction of the descending pain-modulating network^1–3^. Modulating the neural circuitry of ipsilateral PH has been found to have positive outcomes in a variety of neurological conditions, however, there is a lack of information about the effects of the PH on drug-resistant orofacial pain and its possibility as a promising target for trigeminal neuropathic pain (TNP) management.

Abnormalities in the afferent neurons of the trigeminal nerve and ganglion instigate TNP. Although there have been meaningful advancements in the understanding and management of TNP in recent decades, most of the present therapeutic approaches cannot provide long-lasting relief from the condition. This situation necessitates new substitute therapeutic approaches^4^. Since TNP is governed by the activity of nociceptors via the trigeminal ganglion (TG) and trigeminal nucleus caudalis (TNC) and PH has major projections to both the vlPAG (direct) and to TNC (via the trigemino-hypothalamic fibers), altering the activity of the PH could provide novel insight into TNP management^5,6^. Drug refractory TNP may involve any of the trigeminal branches and alter the sensory transmissions. However, atypical trigeminal pain involves the second and third branches of the trigeminal nerve predominantly. The infraorbital nerve (ION), which is originated from the second branch of the trigeminal nerve and is a pure sensory nerve in rodents, constitutes majority of the maxillary division of the trigeminal nerve and plays important role in facial tactile and pain sensation. ION comprises mystacial vibrissae, vibrissal pad, part of rhinarium, upper teeth, and part of the dorsal oral cavity. Therefore, injury to ION influences quantitative alterations in nonevoked behavioral activity as well as in responses induced by mechanical stimulation of the facial area^7–10^. Thus, chronic constriction injury of the infraorbital nerve (CCI-ION) of rodents is a well-established model for studying the sensory alterations related to TNP because sensory abnormalities are directly linked to alterations in the ION, and CCI-ION animal model exhibits most common TNP symptoms^4,7,11^.

**Figure 1:**
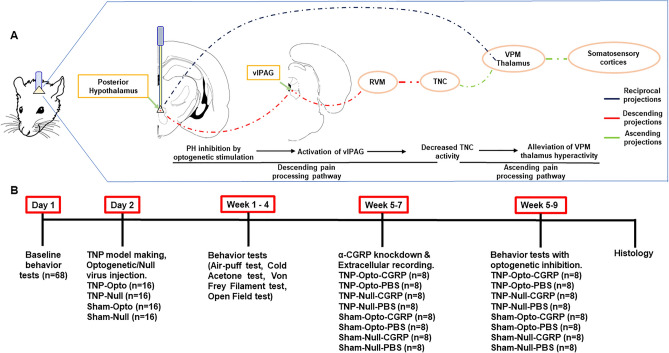
Targeted neural circuitry and experimental timeline. (**A**) Modulation of descending pain processing pathway by optogenetic inhibition of PH. (**B**) Experimental timeline. PH = Posterior hypothalamus; vlPAG = Ventrolateral periaqueductal gray; CCI-ION = Chronic constriction injury of infraorbital nerve; RVM = Rostral ventromedial medulla; TNC = Trigeminal nucleus caudalis; VPM thalamus = Ventral posteromedial thalamus.

Since the altered neural activity of PH is a mechanism found to be involved in trigeminal pain disorders including TACs^12^, migraine^13^, and TN^14^, the objectives of this study were to observe the involvement of PH in TNP and the outcomes once we optogenetically inhibited the activity of the PH in a CCI-ION rat model. Figure 1A,B show the neural circuitry involved in our experiment and our experimental timeline.

## Results

### Behavioral pain responses following CCI-ION surgery

In our study, CCI-ION surgery was performed to generate both spontaneous and evoked nociceptive behaviors similar to TNP condition. All the animals in the TNP group showed significant pain responses in every behavioral test after ION ligation, whereas sham and control animals did not exhibit any significant alterations in behavioral test scores.

**Figure 2:**
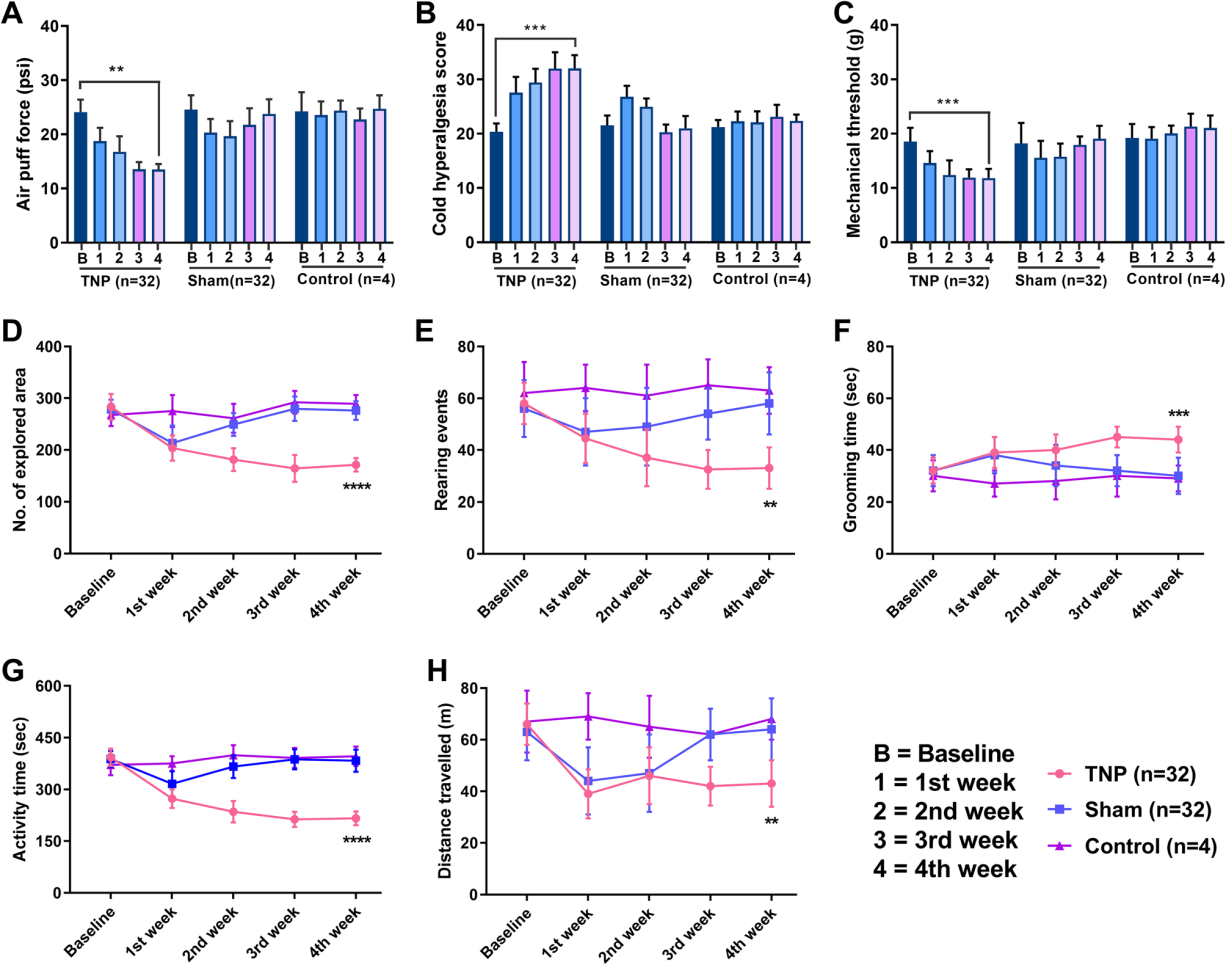
Trigeminal pain behavioral scores after CCI-ION surgery. (**A**) Results from the air-puff test on the ipsilateral trigeminal facial area. (**B**) Cold allodynia score results for the ipsilateral facial area with acetone drops. (**C**) Mechanical allodynia (von Frey test) results for the ipsilateral facial area. (**D**–**H**) Results of the open field test: (**D**) number of explored areas, (**E**) number of rearing events, (**F**) duration of grooming time, (**G**) duration of active time, and (**H**) distance traveled. **, *p* < 0.01; ***, *p* < 0.001; ****, *p* < 0.0001, repeated measures one-way ANOVA. Data are displayed as means ± SD.

A significant decrease in the air-puff test scores of TNP animals over the four weeks (24.11 ± 2.31 psi to 13.51 ± 1.02 psi; repeated measures one-way ANOVA, *F* (1, 7) = 211.28, *p* < 0.01; Fig. 2A) was observed. The facial cold hyperalgesia test showed significant increase (20.34 ± 1.54 to 32.02 ± 2.43; repeated measures one-way ANOVA, *F* (1, 7) = 314.17, *p* < 0.001; Fig. 2B) after surgery. The mechanical allodynia scores also significantly decreased (18.55 ± 2.54 g to 11.82 ± 1.69 g; repeated measures one-way ANOVA, *F* (1, 7) = 302.07, *p* < 0.001; Fig. 2C) following surgery.

After analyzing the results in the open field test using repeated measures one-way ANOVA, TNP animals showed significant alterations in exploration rate (*p* < 0.0001, Fig. 2D), rearing events (*p* < 0.01, Fig. 2E), grooming time (second) (*p* < 0.001, Fig. 2F), active time (second) (*p* < 0.0001, Fig. 2G) and overall distance traveled (meter) (*p* < 0.01, Fig. 2H) compared to the sham and control group animals (Supplementary Table 1). All these results indicated that ION ligation induced allodynia and hypersensitive behaviors in TNP animals.

### Optogenetic inhibition alters vlPAG neuronal firing activity

To observe the alterations of vlPAG neural activity in CCI-ION condition and under the influence of PH inhibition condition, single-unit extracellular recordings were performed, and different electrophysiological traits were examined. All the TNP and sham animals randomly received either shCGRP injection or, PBS injection into the trigeminal ganglion before recording and were divided into TNP-Opto-shCGRP (n = 8), TNP-Opto-PBS (n = 8), TNP-Null-shCGRP (n = 8), TNP-Null-PBS (n = 8), Sham-Opto-shCGRP (n = 8), Sham-Opto-PBS (n = 8), Sham-Null-shCGRP (n = 8) and Sham-Null-PBS (n = 8) groups.

**Figure 3:**
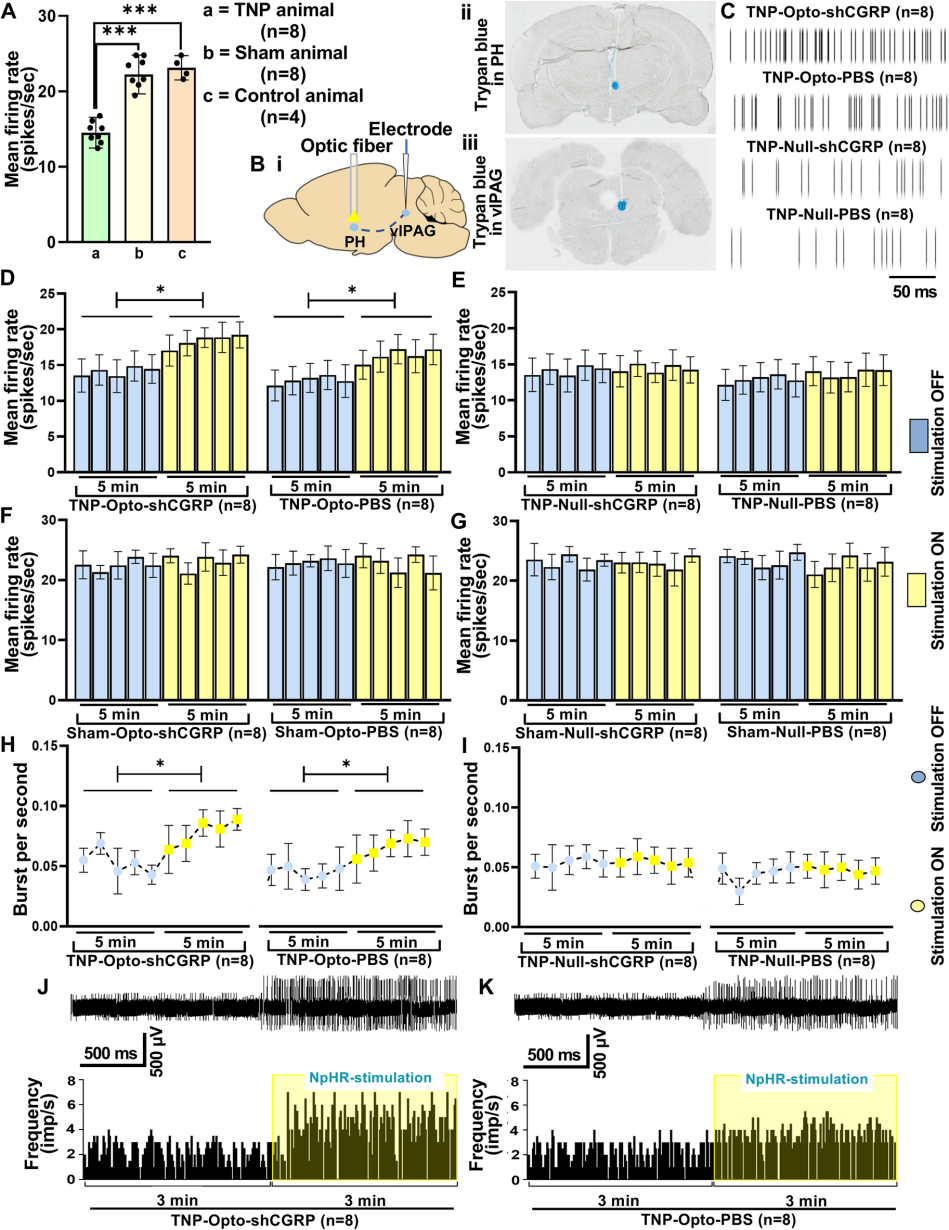
In vivo extracellular recording from the vlPAG. (**A**) Spontaneous firing rates of TNP (*n* = 8) animals compared to sham (*n* = 8) and control (*n* = 4) animals (***, *p* < 0.001, unpaired *t tests*). (**B**) Diagram showing the optogenetic inhibition and extracellular recording areas (i), trypan blue confirming the optic fiber tip position in the PH (ii) and the electrode tip position in the vlPAG (iii). (**C**) Recorded representative raster traces of the time calibration of 50 ms from the vlPAG of the TNP-Opto (n = 16) and TNP-Null (n = 16) groups. (**D**–**G**) Spontaneous neural firing rates of the vlPAG in the TNP-Opto (n = 16) (D), TNP-Null (n = 16) (**E**), Sham-Opto (n = 16) (**F**), and Sham-Null (n = 16) (**G**) groups. (**H**,**I**) Burst firing rates of the vlPAG in the TNP-Opto (n = 16) (**H**) and TNP-Null (n = 16) (**I**) groups. (**J**,**K**) Representative example of the increase in instantaneous firing frequency caused by optic stimulation in the TNP-Opto-shCGRP (n = 8) (**J**) and TNP-Opto-PBS (n = 8) (**K**) groups.

In vivo extracellular recording from the vlPAG exhibited significantly reduced firing rates in the TNP rats (n = 8) (14.52 ± 2.04 spikes/s) compared to that of the sham (n = 8) (22.23 ± 2.57 spikes/s, unpaired *t test* (t, df) = (6.646, 14), *p* < 0.001) and control (n = 4) rats (23.13 ± 1.61 spikes/s, unpaired *t test* [t, df] = 5.49, 10), *p* < 0.001) (Fig. 3A).

We observed the effects of optogenetic inhibition of the PH on the neuronal activity of the vlPAG (Fig. 3B). An increased frequency of representative raster traces was observed in the TNP-Opto group compared to the TNP-Null group (Fig. 3C). Inhibition of the PH resulted in a significant increase in vlPAG neuron activity in the TNP-Opto group. The spike rates in the stimulation-OFF and stimulation-ON conditions of the TNP-Opto-shCGRP (n = 8) group were 14.124 ± 2.17 and 18.42 ± 1.85, respectively (two-way ANOVA, *F* (1, 70) = 5.786, *p* < 0.05; Fig. 3D), and those of the TNP-Opto-PBS (n = 8) group were 12.91 ± 2.1 and 16.83 ± 2.14, respectively (two-way ANOVA, *F* (1, 70) = 5.68, *p* < 0.05; Fig. 3D). No significant changes in the vlPAG firing rates were observed in the TNP-Null (Fig. 3E), Sham-Opto (Fig. 3F), and Sham-Null groups (Fig. 3G). The burst firing rates were also increased in the vlPAG of the TNP-Opto group. The burst rates for the stimulation-OFF and stimulation-ON conditions in the TNP-Opto-shCGRP (n = 8) group were 0.053 ± 0.011/s and 0.077 ± 0.014/s, respectively (two-way ANOVA, (*F* (1, 70) = 5.284, *p* < 0.05; Fig. 3H) and in the TNP-Opto-PBS (n = 8) group were 0.045 ± 0.013/s and 0.065 ± 0.014/s, respectively (two-way ANOVA, *F* (1, 70) = 5.03, *p* < 0.05; Fig. 3H). There were no significant alterations in the burst rate of the TNP-Null animals (Fig. 3I). Increased instantaneous firing frequency of the vlPAG in response to optogenetic inhibition of the PH in both the TNP-Opto-shCGRP (Fig. 3J) and TNP-Opto-PBS (Fig. 3K) groups was observed under stimulation-ON condition.

These findings indicated that inhibition of PH can restore the altered neural activity of vlPAG in TNP rats.

### Optogenetic inhibition alters VPM thalamic firing activity

**Figure 4:**
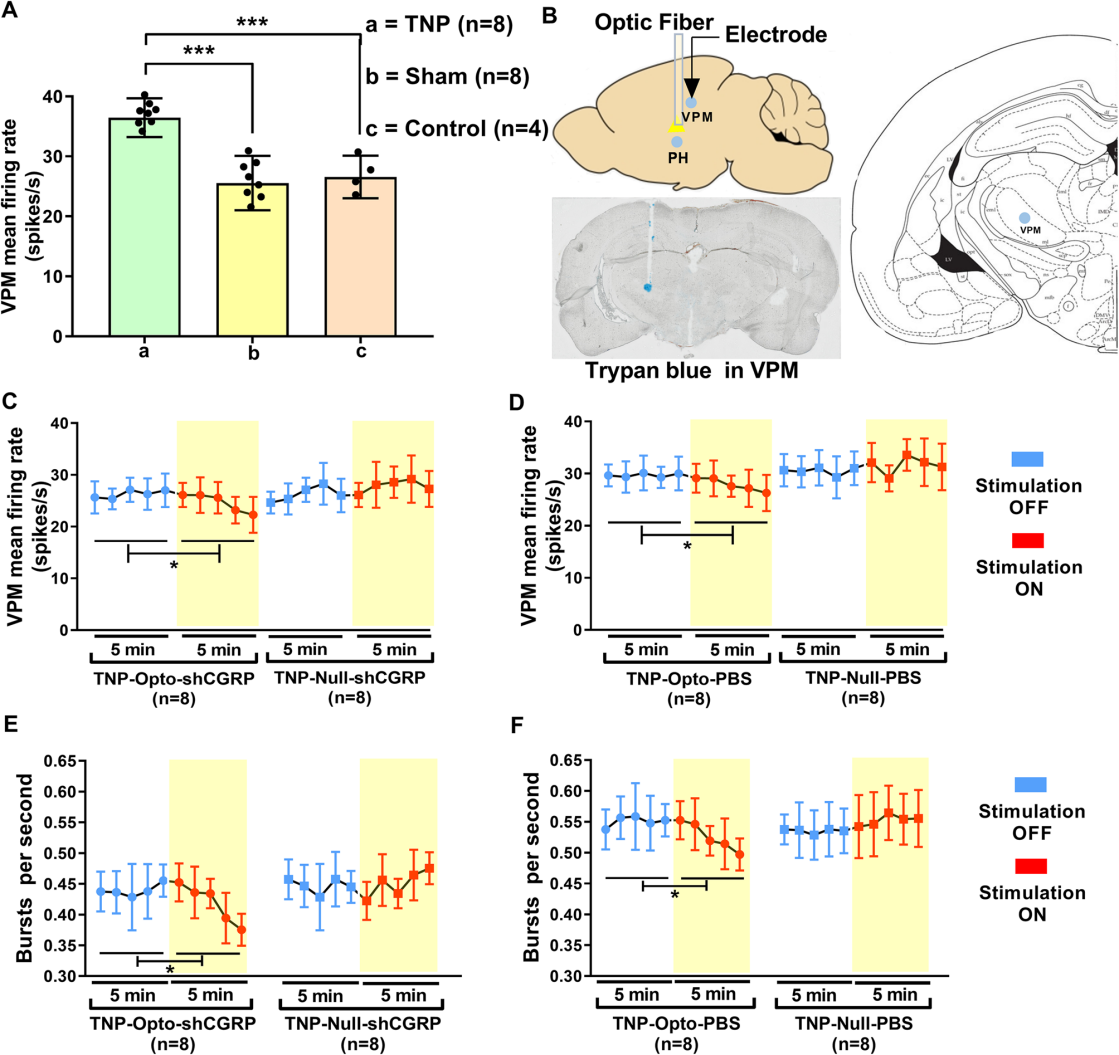
In vivo extracellular recording from VPM thalamus. (**A**) Spontaneous firing rates of TNP (*n* = 8) animals compared to sham (*n* = 8) and control (*n* = 4) animals (***, *p* < 0.001, one-way ANOVA). (**B**) Schematic diagram showing the extracellular recording area. (**C**,**D**) In vivo recordings of the VPM thalamus from TNP-Opto-shCGRP (n = 8), TNP-Null-shCGRP (n = 8) (**C**); TNP-Opto-PBS (n = 8) and TNP-Null-PBS (n = 8) (**D**) groups. A decrease in firing output (spikes/s) was observed in both TNP-Opto groups during the stimulation “ON” period. (**E**,**F**) Changes in the burst firing rates of TNP-Opto animals during optogenetic stimulation. A significant reduction was observed in the TNP-Opto-shCGRP (n = 8) (**E**) and TNP-Opto-PBS (n = 8) (**F**) groups. *, *p* < 0.05, repeated measure one-way ANOVA.

We also explored the neural activity of VPM thalamus in both pain and optogenetic stimulation conditions to identify if PH inhibition could alter the hyperactivity of thalamic neurons. During single unit recording from VPM neurons in different animal groups of our study, we observed a significantly increased thalamic firing rate in the TNP (n = 8) rats (36.435 ± 3.233 spikes/s) compared to the sham (n = 8) (25.563 ± 4.534 spikes/s, one-way ANOVA, *F* (2, 17) = 10.87, *p* < 0.001) and control (n = 4) rats (26.564 ± 3.563 spikes/s, one-way ANOVA, *F* (2, 17) = 10.19, *p* < 0.001) (Fig. 4A).

After analyzing the electrophysiology data with PH inhibition (Fig. 4B), we detected significant reduction in VPM neuron activity in the TNP-Opto group. The spike rates in the stimulation-OFF and stimulation-ON conditions of the TNP-Opto-shCGRP (n = 8) animals were 26.28 ± 2.74 and 24.65 ± 2.97, respectively (Repeated measure one-way ANOVA, *F* (1.755, 7.018) = 11.43, *p* < 0.05; Fig. 4C). The spike rates in the stimulation-OFF and stimulation-ON conditions of the TNP-Opto-PBS (n = 8) animals were 29.68 ± 2.74 and 27.85 ± 3.05, respectively (Repeated measure one-way ANOVA, *F* (1.292, 5.169) = 10.29, *p* < 0.05; Fig. 4D). The TNP-Null groups showed no significant alterations in neural firing rate (Fig. 4C,D). Burst firing rates of the VPM thalamus in the stimulation-OFF and stimulation-ON conditions of the TNP-Opto-shCGRP animals (n = 8) were 0.44 ± 0.04/s and 0.41 ± 0.03/s, respectively (Repeated measure one-way ANOVA, *F* (1.658, 6.631) = 9.31, *p* < 0.05; Fig. 4E), and in the TNP-Opto-PBS animals (n = 8), they were 0.55 ± 0.03/s and 0.52 ± 0.03/s, respectively (Repeated measure one-way ANOVA, *F* (1.538, 6.152) = 8.94, *p* < 0.05; Fig. 4F). There were no changes detected in the burst firing rate of the TNP-Null animals.

These data implied that inhibition of PH can modulate the increased VPM thalamic firing activity in TNP animals.

### Improvement of behavioral responses with optogenetic inhibition of the PH

**Figure 5:**
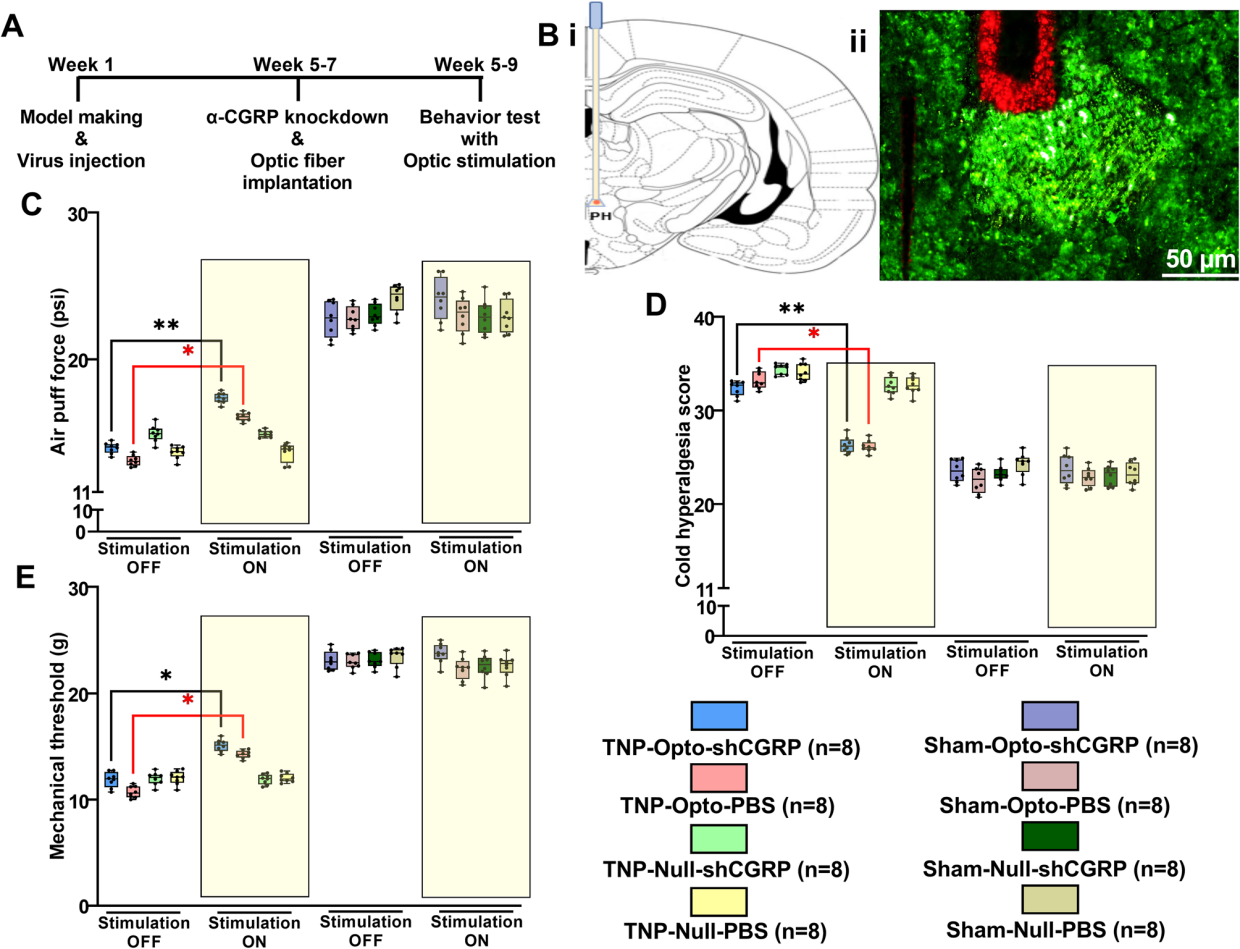
Optogenetic inhibition of PH improved TNP pain behavioral responses. (**A**) Timeline of optogenetic virus injection, optic fiber implantation and behavior tests. (**B**) Schematic diagram showing the location of the PH (i) and representative images of the optic fiber implantation location in the PH (ii). Positive expression of NpHR-EYFP was observed in the PH (green) and GFAP immunostaining (red) illustrated the optic fiber tract to the PH. Scale bar: 50 µm. (**C**–**E**) Changes in behavioral responses following yellow laser stimulation. The results from the air-puff test (**C**), cold allodynia score test (**D**), and von Frey test (**E**). The TNP-Opto groups exhibited significant changes in behavioral scores during the stimulation “ON” condition. No significant changes in behavioral scores were observed in other animal groups during the stimulation “ON” condition. *, *p* < 0.05; **, *p* < 0.01, significant differences determined using paired *t*-test. Data are displayed as means ± SD.

After electrophysiology and optic fiber cannula implantation, all behavioral tests were performed again, with and without optogenetic stimulation to observe whether PH inhibition improved the pain behavior responses (Fig. 5A,B). All the animals were kept with intermittent yellow laser stimulation-ON condition from three minutes prior to performing the tests and then the stimulation remained ON during the total duration of the behavior test. Significant improvement in the behavioral responses of TNP animals was noticed during PH inhibition condition.

**Figure 6:**
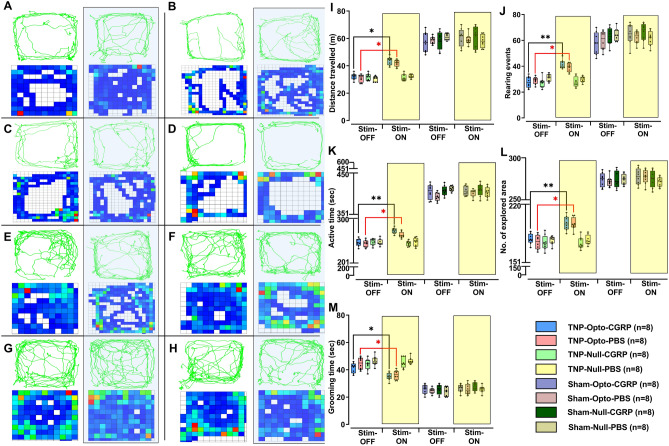
Alteration of behavioral responses in the open field test upon optogenetic inhibition. (**A**–**H**) Trajectory and exploration representations of the TNP-Opto-shCGRP (n = 8) (**A**), TNP-Opto-PBS (n = 8) (**B**), TNP-Null-shCGRP (n = 8) (**C**), TNP-Null-PBS (n = 8) (**D**), Sham-Opto-shCGRP (n = 8) (**E**), Sham-Opto-PBS (n = 8) (**F**), Sham-Null-shCGRP (n = 8) (**G**), and Sham-Null-PBS (n = 8) (**H**) groups during stimulation “OFF” and “ON” conditions. (**I**) Distance traveled, (**J**) number of rearing events, (**K**) duration of active time, (**L**) number of explored areas, and (**M**) duration of grooming time. In all the behavioral paradigms, the TNP-Opto-shCGRP (n = 8) and TNP-Opto-PBS (n = 8) groups exhibited significant alterations in the stimulation “ON” condition but no significant changes in behavioral scores were observed in other animal groups in the stimulation “ON” condition. *, *p* < 0.05; **, *p* < 0.01, significant differences determined using paired *t*-test. Data are displayed as means ± SD.

Improvement in behavioral responses were found in the air-puff test during the stimulation-ON condition in both the TNP-Opto-shCGRP (n = 8) group (13.98 ± 1.75 psi to 17.38 ± 1.84 psi, paired *t test* (t, df) = (8.82, 14), *p* < 0.01; Fig. 5C) and in the TNP-Opto-PBS (n = 8) group (13.11 ± 1.69 psi to 16.08 ± 1.86 psi, paired *t test* (t, df) = (7.619, 14), *p* < 0.05; Fig. 5C). Cold hyperalgesia scores also showed improvement with stimulation-ON condition in the TNP-Opto-shCGRP (n = 8) group (32.38 ± 2.14 to 26.29 ± 1.86, paired *t test* (t, df) = (5.05, 14), *p* < 0.01; Fig. 5D) and in the TNP-Opto-PBS (n = 8) group (33.18 ± 2.05 to 28.03 ± 1.96, paired *t test* (t, df) = (4.771, 14), *p* < 0.05; Fig. 5D). Optogenetic inhibition of PH improved the mechanical allodynia scores in the TNP-Opto-shCGRP (n = 8) group (11.86 ± 1.69 g to 14.079 ± 1.53 g, paired *t test* (t, df) = (2.79, 14), *p* < 0.05; Fig. 5E) and in the TNP-Opto-PBS (n = 8) group (10.71 ± 1.51 g to 13.88 ± 1.34 g, paired *t test* (t, df) = (2.175, 14), *p* < 0.01; Fig. 5E). Optogenetic inhibition of the PH also resulted in significant improvement of behavioral scores in all the analyzed parameters of the open field test (OFT) in TNP-Opto animals (Supplementary Table 2 and Fig. 6).

Although optogenetic inhibition of PH resulted in improved behavioral responses in both CGRP knockdown and active state, CGRP knockdown condition with optogenetic stimulation exhibited to have a more robust effect on behavioral responses of TNP animals (Supplementary Table 3). However, no significant changes in behavior were observed during optogenetic inhibition in the other animal groups (TNP-Null, Sham-Opto, and Sham-Null). These results indicated that inhibiting PH caused anti-nociceptive effect in TNP rats.

### Immunofluorescence results

**Figure 7:**
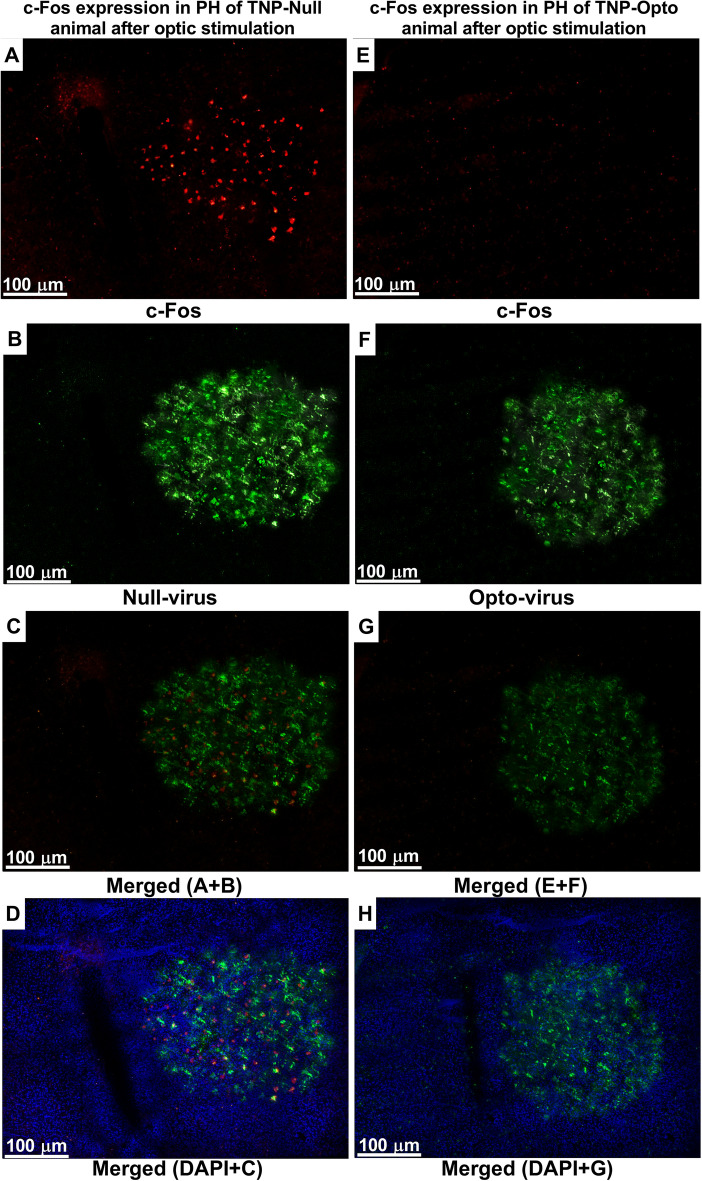
c-Fos expression in the PH of null virus- and opto virus-injected TNP animal after optogenetic stimulation. (**A**–**D**) c-Fos and null virus' expression in the PH of TNP-Null animals: (**A**) expression of c-Fos-positive neurons, (**B**) Null virus’ expression in PH, (**C**) merged (c-Fos + Null-virus), (**D**) merged (c-Fos + Null-virus + DAPI). (**E**–**H**) c-Fos and opto virus’ expression in the PH of TNP-Opto animals: (**E**) alleviated c-Fos expression, (**F**) Opto virus’ expression in PH, (**G**) merged (c-Fos + Opto-virus), (**H**) merged (c-Fos + Opto-virus + DAPI). Scale bar = 100 µm.

The TNP animals showed increased c-Fos expression in PH, indicating that PH had become hyperactive following CCI-ION surgery (Supplementary Fig. 1A–C). Sham animals, on the other hand, showed no or very little c-Fos expression in the PH (Supplementary Fig. 1D–F). To observe the expression of optogenetic virus and to confirm that our injected optogenetic virus caused inhibition of PH after yellow laser stimulation, we examined colocalization of c-Fos expression and optogenetic virus expression in the PH of TNP animals because activated neural area exhibits increased c-Fos expression^4^. Immediately after performing behavior tests with optogenetic stimulation-ON condition, the animals were euthanized, and brain tissue slides were prepared for immunofluorescence staining. In TNP-Null group animals, we found colocalization of activated c-Fos expression and null virus expression in PH (Fig. 7A–D) indicating increased immunoreactivity in the PH of TNP animals in response to trigeminal pain. Whereas, in TNP-Opto group animals, we observed reduced c-Fos expression colocalizes with optogenetic virus expression (Fig. 7E–H) in the PH. We also observed positive staining of somatostatin (Supplementary Fig. 2A–C) neurons in our targeted area.

In the TG, active CGRP expression colocalized with substance P was found in animals injected with PBS (Supplementary Fig. 3A–D), whereas CGRP expression was not observed in animals injected with shCGRP (Supplementary Fig. 3E–H). In quantification analysis of presence of active CGRP in TG (Supplementary Fig. 3I), 68.83 ± 6.35% and 26.76 ± 4.81% (unpaired *t* test) of CGRP expression were observed in TNP-PBS (n = 8) and TNP-CGRP (n = 8) group animals, respectively; and 38.07 ± 3.12% and 21.32 ± 5.97% (unpaired *t* test) of CGRP expression were observed in Sham-PBS (n = 8) and Sham-CGRP (n = 8) group animals, respectively. Supplementary Fig. 4 showed optogenetic and null virus’ expression in PH with different magnifications.

## Discussion

The optogenetic approach, in association with extracellular recording, is a useful method to examine selective neural circuit function and determine how specific neuron types contribute to behavior. In our study, we applied optogenetic inhibition to the PH of a CCI-ION rat model in both with or without α-CGRP knockdown condition and observed behavioral improvement in different behavioral tests as well as in neuronal firing during stimulation. Taken together, these results suggest that inhibition of PH activity has anti-nociceptive effects on trigeminal neuropathic pain by modulating the trigeminal pain processing pathway.

### Altered pain threshold levels in TNP

In TNP, nociceptive information, which can be triggered by several factors such as nerve compression by different anomalies, nerve trauma, nerve demyelination due to infectious agents etc., relayed through the spinal TNC and deterioration of the protective myelin sheath results in an inconsistent and hyperactive nerve state, that leads to pain from even a minimal stimulation of any area innervated by the nerve and hinders the cessation of pain signals after the stimulation has ended^6,15^. After the CCI-ION surgery, we also observed that TNP animals exhibited lower pain threshold levels in different behavioral tests than the animals in the sham and control group.

### Involvement of PH in TNP

During TNP different parts of the brain are involved in both the ascending and descending pain processing pathways and the activity of local neural circuitry is either increased or decreased to aid in the transmission of pain signals. Of the involved regions, the PH, which is a part of the descending pain modulation network, is one of the crucial areas that is directly connected with and has a significant impact on neurophysiological circuits involved in pain sensations, specifically in the trigeminal pain processing pathway^14,16^. PH receives nociceptive information from the VPM thalamus by trigeminovascular inputs and electrical stimulation of PH showed modulatory effect on thalamic region which indicated the presence of bidirectional connection between PH and thalamus. PH stimulation also produced alterations in the activity of somatosensory cortex, anterior cingulate cortex, middle temporal gyrus, insula and all these brain areas are part of the pain matrix. The effect of PH on these areas suggested the involvement of PH in top-down modulation^17–19^. PH has also a reciprocal connection with TNC via the trigemino-hypothalamic tract where the sensory information’s from regions involved in the trigeminal system (i.e. cranial skin, meninges) enter the PH and alteration of the PH activity affects the activity of TNC neurons as well^19,20^. Available evidence suggests that the PH is crucial in the generation of nociception in various TNP conditions^16,19,21^. Increased neuronal activity of the ipsilateral PH local circuitry has been observed in PET neuroimaging studies, during chronic trigeminal pain and other trigeminal autonomic cephalalgias^1,2,14,22,23^. Similar to previous studies, we also observed that facial nociceptive stimulation elevated Fos immunoreactivity of the PH after CCI-ION surgery which indicated enhanced activation of PH^21,24,25^.

### Precise neuromodulation by optogenetic stimulation

Currently, no therapeutic approaches have been designated as a long-term treatment method for TNP management. Several techniques, such as stereotactic radiosurgery, radiofrequency (RF) lesions, and microvascular decompression (MVD), have been able to reduce TNP on the basis of the main reason behind it; however, the pain recurred in most patients^15,23,26^. Bearing that in mind, nowadays different neurostimulation techniques have exhibited promise as treatment modalities, and researchers are hopeful that neurostimulation approaches could assist in discovering new breakthroughs in TNP management^27,28^. Among the existing neurostimulation techniques, the optogenetic approach is a revolutionary technology in the neuroscience field that allows remarkable precision in understanding different neurodevelopmental processes and observing how modulation of neuronal circuitry results in behavioral variation^29,30^.

### PH-inhibition induced modulatory effects in TNP

The PH participates in neurovegetative feedback associated with the pain threshold of the ipsilateral orbital region, cortical excitability, and behavioral responses^14^. Any of the trigeminal divisions could be involved in TACs and TNP, with the same areas of pain and variations in clinical presentations^15,31,32^. Several human and animal studies have found that alteration of the ipsilateral PH activity with the aim of inhibiting hyperactive neurons results in a reduction in pain in TAC and TNP patients^14,16,22,23,25,33–35^. However, all previous studies used electrical stimulation of PH in trigeminal neuropathies, and it was unclear whether the stimulation increased or decreased PH activity because electrical stimulation can cause both depolarization or hyperpolarization of the neuron, and whether the neurons will be excited or inhibited depends on the distance of neuron from the stimulation source as well^36^. As a result, since our aim was to observe the results of inhibiting the activated neurons of PH in TNP condition, we adopted an optogenetic inhibition strategy in our investigation to get selective modulation of PH activity and our behavioral results were in line with our prediction, revealing elevated pain threshold levels during PH inhibition in both with or without CGRP knockdown condition.

Effect of PH inhibition acts through the remodeling of neural circuits involving the vlPAG- and RVM-mediated endogenous descending pain pathway in the brain and nociceptive inputs to the TNC can be altered by modulating the PH due to the projections from the PH to the TNC^1,5,14,16,17,21,24,34,37–39^. Synaptic activity of the anti-nociceptive projections from the PH is robustly mediated by GABA^16,34^, and the vlPAG receives robust projections from the PH^5^, which plays an important role in trigeminal nociception modulation. In the context of noxious stimuli, GABAergic neurotransmission in the vlPAG is pronociceptive^39^. Therefore, inhibiting PH activity in TNP, ultimately results in increased vlPAG activity, may provide a modulatory effect on the complex neural circuitry involved in trigeminal pain processing. During neuronal recording, we also found increased vlPAG activity in TNP animals under the influence of PH inhibition which is consistent with studies where trigeminal nociception was inhibited by PAG stimulation^3,5,17,34,39,40^.

In addition to recordings in the vlPAG, we also performed extracellular recordings in the VPM thalamus. Sensory pain originates within the spinal dorsal horn and ascends via the spinothalamic pathway to terminate within the thalamus. The trigeminal pain pathways synapse on tertiary neurons of the VPM thalamus, which in turn relays information to the primary and secondary somatosensory cortices. Therefore, increased VPM thalamus activity is well documented in TNP^4,6,11^, and we observed similar effects in our study after CCI-ION surgery which were later ameliorated by PH inhibition.

### Limitations

This study had a few limitations. For example, we only included female animals in our study. Postoperative stress might have affected measurements of chronic pain. We did not record neural firing activity from the PH itself, and anesthesia might have also influenced the neural firing rates, although we provoked ipsilateral whisker pads with von Fray filament to avoid such influence. Scrambled shRNA injection into the TG would have been a more appropriate control than PBS. Neural activity of vlPAG and VPM in post-stimulation condition was not assessed.

## Conclusion

To recapitulate, inhibiting PH activity in TNP has anti-nociceptive effects on TNP animal model. Although clinical application of optogenetic approach still has a long way to go, but the target specificity of such strategies provides efficient knowledge about the underlying mechanism. Our study showed that inhibition of PH results in alteration of both ascending and descending pain pathway projections which may offer unprecedented potential insights in therapeutic management of TNP.

## Methods

### Experimental subjects and ethics approval

Sixty-eight female Sprague–Dawley rats (age: 8 weeks; Koatech, Pyeongtaek, Korea) were used in this study, each weighing 200–250 g at arrival. All animals were kept in a room with a 12:12 h light and dark cycle, a constant temperature (22 ± 1 ºC), and humidity (50–60%) and had ad libitum access to fresh water and food. All surgical procedures were performed under general anesthesia with an intraperitoneal injection of 15 mg/kg Zoletil (Zoletil50®, Virbac Laboratories, Carros, France) and 9 mg/kg Rompun (Rompun®, Bayer, Seoul, South Korea) and the animals were monitored afterward. All experiments were in strict accordance with the guidelines of Chungbuk National University Laboratory Animal Research Center, the experiment protocols were approved by Chungbuk National University's Animal Care and Use Committee (IACUC) (CBNUA-1346-20-02) and the reporting in the manuscript follows the recommendations in the ARRIVE guidelines. To alleviate the animals' suffering, they were handled with care before, during and after the experiments. Only female animals were used in our study since it is widely known that the prevalence of trigeminal nerve injury related pain is higher in females^41,42^.

### CCI-ION model and optogenetic virus injection

CCI-ION surgery was performed on 32 animals to generate the TNP model after the baseline test, as described previously^43^ (Supplementary Method 1). Briefly, after the rats were given general anesthesia, two ligatures made of 3–0 silk suture were placed 3–4 mm apart on the revealed ION. Finally, silk sutures (3–0) were used to close the incision above the eye of the TNP group animals (n = 32). The same procedure was performed on sham group animals (n = 32) except for the ligature placement.

To acquire the desirable control over PH, we used optogenetic neurostimulation approach in our study. Therefore, TNP and sham group animals were randomly divided into two subgroups (TNP-Opto, TNP-Null and Sham-Opto, Sham-Null; 16 animals in each group) immediately after the CCI-ION surgery and the Opto groups were injected with an optogenetic virus and the Null groups were injected with a null virus. We used AAV-hSyn-eNpHR3.0-EYFP (1.9 × 10^13^ GC/µL; Korea Institute of Science and Technology, Seoul, South Korea) as the optogenetic virus for the inhibition of PH activity and AAV-hSyn-EYFP (5 × 10^12^ GC/µL; Korea Institute of Science and Technology, Seoul, South Korea) as the null virus. The optogenetic or, null viral vector was injected in the ipsilateral PH (AP, bregma, -3.7 mm; ML, midline, -0.3 mm; DV, skull, -7.7 mm) of the CCI-ION side with a stereotaxic apparatus. Each animal was administered 2 µL of the virus. The Hamilton syringe was held in the same spot for 5 min after the injection and then gradually retracted. To confirm our targeted area, we stained our brain tissue sections with somatostatin antibody because somatostatin neurons are found in the PH^34^. After the surgery, the animals were placed back in their cages and monitored daily for at least one week.

### Behavioral assessment for pain response

All behavioral tests were carried out by an examiner who was blind to the groups. Behavior tests were performed one day prior (baseline) to CCI-ION surgery and at every seventh day afterward up to the fifth week. All the experiments were randomized and performed in two days’ period every time. One day we performed air-puff test and cold hyperalgesia test and in the next day we performed von-Fray filament test and open field test. At week eight after the optic fiber implantation, all the behavior tests were performed once again with optic stimulation OFF and ON condition to assess the effect of optogenetic inhibition of ipsilateral PH. In all the behavior tests, data from each animal was taken three times and then the mean data was recorded as behavioral score.

The air-puff test was carried out to assess the mechanical allodynia of the ipsilateral orofacial side to the CCI-ION, according to identical protocols as earlier studies^11^. In brief, the rats were acclimatized in a rodent Panlab holder (Scitech Korea Inc., Seoul, South Korea) in a dark, noise-free environment for 30 min and then a constant blow of air for four seconds was given to the injured side of the orofacial area with a ten seconds of interval period every time. The pound-force per square inch (psi) level of the air-puff was started a 5 psi and the psi level where the animal started to rub their ipsilateral facial side aggressively or, biting the air tube tip was recorded.

For the cold hyperalgesia response assessment, the animals were kept in the plexiglass cage for 30 min and then a glass syringe was used to apply a few drops of acetone (it decreases the skin temperature and then evaporates quickly as well as it incorporates not only an innocuous cold stimulus but also mechanical and chemical stimulations) to the ipsilateral vibrissal pad of the CCI-ION^11^. Then the total number of reflexive behaviors (scratching/rubbing the ipsilateral facial side) over the next 2 min was recorded and counted as cold hyperalgesia score. Only the face was included in the assessment.

To determine the mechanical threshold level of the orofacial region, von Frey filaments were used. After a 30 min of habituation period where the filaments were brought near to the animals at every five minutes interval for a few seconds, the stimuli were delivered near the center of the vibrissal pad on the ipsilateral CCI-ION side of the animal by using the filaments^11^. We started from 0.6 gm and gradually increased the force level with 15 s interval every time. The lowest force required to produce painful or aggressive reactions (quick withdrawal, escape behaviors, attacking the filament, or asymmetric face stroking) was then recorded.

In the open field test, animals were habituated in the test room for 30 min and then placed at a corner of a plain, illuminated 70 cm × 70 cm × 30 cm arena that was cleaned with 95% ethanol. Then the responses of the animals were recorded for 10 min with an overhead video camera that was set vertically 1 m above the test field. Afterward, the number of explored locations, number of rearing events, active time (second), overall distance traveled (meter), and pain-like behavior (facial grooming) (second) were analyzed from the video recordings^4^. ToxTrac software was used to track and analyze all the individual behavior parameters^44^.

### α-CGRP knockdown in the trigeminal ganglion

In our study, the CGRP knockdown technique was used as a positive control to observe the effect of optogenetic inhibition on behavioral responses in both CGRP active and knockdown conditions since α-CGRP is necessary for the transmission of neuropathic hypersensitivity. In the trigeminal system, CGRP is the most significant neuropeptide that shows an ascendant existence in TRPV1-expressing sensory neurons as α-CGRP. Furthermore, researchers have found that anti-CGRP antibodies and CGRP receptor antagonists exhibited favorable effects in trigeminal system^45^. Therefore, all the animals from the TNP-Opto, TNP-Null, Sham-Opto, and Sham-Null groups were again divided into two subgroups consisting of eight animals, where one subgroup received a shCGRP (AAV2-GFP-U6-r-CRCP-shRNA, 3.8 × 10^12^ GC/mL; Vector Biolabs, Malvern, United States) injection and another subgroup received PBS directly into the ipsilateral trigeminal ganglion (AP: − 3.5 mm, ML: − 3.6 mm, DV: − 12 mm^11^) 30 min prior to electrophysiology recording at week 5–7. A Hamilton syringe and an automated micro-syringe pump were used to inject shCGRP at a rate of 0.3 µL/min and the needle was kept for more five minutes to ensure the absorption of the shCGRP. CGRP knockdown approach had no effect on our neural recording. Due to the ethical concerns regarding the effect of so many surgeries on the animals, we decided to inject shCGRP while performing electrophysiology so that we did not have to perform individual surgery for injecting shCGRP. That is why 30 min prior to electrophysiology, we performed shCGRP injection in the TG. We kept the 30 min break so that the animal could have a minimum time of recovery from the stress of injection into the TG and we could get electrophysiology data without any other influence on the animal’s sensory system.

### Extracellular recording in vivo with optogenetic inhibition of PH

To activate the eNpHR3.0 ion channel, yellow laser light (Model: YL589T3-010FC, 589-nm yellow DPSS laser with fiber-coupled (T3), Shanghai Laser & Optics Century Co. Ltd., Shanghai, China) with a wavelength of 593 nm was delivered intermittently (30 s laser-on periods and 20 s laser-off periods) by a power supply (ADR-700D; ADR, Shanghai, China) with a 10 mW output power for five minutes^46^. A waveform generator (Keysight 33511B-CFG001; Keysight, Santa Rosa, CA, USA) was used to regulate the power of the laser and to allow the frequency and pulse widths of the square pulses to be adjusted for optical inhibition. The frequency was set to 20 Hz, laser’s intensity was set to 10 mW and the pulse width was set to 4 ms. A minimum irradiance of 0.4 mW/mm^2^ was delivered on eNpHR3.0 expressed neurons to make sure the inhibition of spiking under a continuous 593 nm of wavelength light’s illumination^47^. In all extracellular recording situation, evoked (provoking ipsilateral whisker pads with von Frey filaments to avoid anaesthetic influence) neural activity was monitored, both stimulation ‘OFF’ and ‘ON’ conditions were carried out for five minutes and stimulation-OFF condition was performed before stimulation-ON condition every time. Neural spiking data from each animal was taken three times with 2 min of break every time before starting neural recording and then the mean data was recorded as firing rate.

At week five, single unit extracellular recordings were obtained from the ipsilateral ventrolateral PAG (AP − 7.8 mm, ML − 0.4 mm, DV − 5.6 mm^48^) and from the contralateral VPM thalamus (AP − 3.5 mm, ML 2.8 mm, DV − 6 mm^11^) using a quartz-insulated carbon electrode (Cat. No.: E1011-20, Carbostar-1, Kation Scientific, LLC, MN 55414 USA). Once general anesthesia was achieved, recordings of neuronal activity during the stimulation-OFF and stimulation-ON conditions were performed inside a dimly lit Faraday cage. We connected an electronic interface board (EIB-36, Neuralynx, USA) to a 36-channel headstage and preamplifiers and recorded the neuronal signals using a high-density electrophysiology acquisition system that consisted of Digital Lynx 4SX (Neuralynx Inc., Bozeman, MT, USA) along with Cheetah software. We digitized and bandpass filtered neuronal activity at 40 kHz and 0.9–5 kHz, respectively. Spikesort 3D software (Neuralynx Inc., Montana, USA) was used to conduct the recoded data sorting offline. The rate histograms (spikes/s) of the recorded data were analyzed using NeuroExplorer version 4 (NEXTechnologies, Neuralynx Inc., CO, 80906, Montana, USA).

In both stimulation-OFF and stimulation-ON conditions, the activity of the neurons was split into burst rates and overall firing rates using NeuroExplorer software (Neuralynx Inc.). Burst rates were defined as a set of at least three spikes with a maximum interval of 4 ms between spikes and a burst interval of 100 ms. We chose similar interspike interval histograms to compare the groups.

### Optic fiber with guide cannula implantation

After extracellular recording, two anchor screws were fixed to the skull and an optic fiber (200 µm core, 230 µm outer diameter, numerical aperture of 0.48, hard polymer cladding type, Doric Lenses; Québec City, Québec, Canada) was implanted ipsilateral to the CCI-ION to transfer laser pulses to the PH (AP: − 3.7 mm, ML: − 0.3 mm) during the behavioral tests and firmly fixed on skull using dental cement (Ortho-jet Pound Package, Lang Dental, USA) at week eight. To optimize the targeting of the PH, the optic fibers were shortened to a length of 7.6 mm. In two animals, the optic fiber was implanted with guide cannula (CXGF-7; Eicom, Fushimi-Ku, Kyoto, Japan) which assisted to inject trypan blue at the exact place of optic fiber’s tip.

### Tissue processing for immunofluorescence

After the 9th week, the animals were euthanized. Transcardial perfusion was performed with PBS followed by 4% paraformaldehyde/PBS (pH 7.4) (PFA). The brain and TG samples were collected and post-fixed for overnight in 4% PFA and cryoprotected in 30% sucrose solution. Brains were frozen in optimal cutting temperature (O.C.T.) compound (Tissue Tek®, Sakura, USA). The brain and TG samples were sectioned with a microtome (Thermo Scientific, Waltham, MA, USA) at 20 µm and 10 µm, respectively. To observe optogenetic and null virus expression, brain sections were mounted with Fluoroshield (aqueous mounting medium with DAPI, Abcam, ab104139) and coverslipped for examination under a fluorescence microscope. To observe the expression of c-Fos, CGRP, GFAP, and somatostatin, the brain sections were washed with wash buffer (10X Tris-buffered saline) and then blocked with blocking solution (10% goat serum albumin) for 1 h. Then, the sections were incubated at 4 °C with a primary antibody diluted at recommended ratio (c-Fos, Abcam ab208942 (1:1000); CGRP, Abcam ab81887 (1:50); GFAP, Abcam ab68428 (1:1000); somatostatin, Abcam ab111912 (1:500); substance P, Abcam ab14184 (1:500)) overnight. All the primary antibodies were diluted in the appropriate ratio recommended by the manufacturer. The next day the sections were washed with wash buffer and incubated with an appropriate secondary antibody (Alexa Fluor 488, ab150113 (1:500), ab150077 (1:500), ab150157 (1:500)) at room temperature for 2 h. After that, the slides were washed again with wash buffer and finally mounted with Fluoroshield and coverslipped.

All the images were captured by cellSens standard software (Olympus Corp., Tokyo, Japan). Merging and quantification of the immunofluorescence pictures were done by using ImageJ software (National Institutes of Health, MD, USA).

### Statistical analysis

We analyzed our data with GraphPad Prism software (version 8.4.2, Inc., San Diego, CA, USA) and it is displayed as the means ± standard deviation. For comparing two groups. Student’s *t*-test or a paired *t*-test was performed. Whereas, for analyzing the comparison among three or more groups, one- or two-way analysis of variance (ANOVA) followed by Tukey’s post hoc test or, repeated-measures ANOVA based on the experimental conditions were performed. Unpaired *t test* was used to compare the quantification of immunofluorescence images. A *p* value of < 0.05 was considered statistically significant in every analysis.

## Supplementary Information

Supplementary Information.

## Data Availability

The datasets used and/or analyzed during the current study are available from the corresponding author on reasonable request.
