# Supplementary material for: Modulation of trigeminal neuropathic pain by optogenetic inhibition of posterior hypothalamus in CCI-ION rat
Source: Sci Rep. 2023 Jan 10;13:489. doi: 10.1038/s41598-023-27610-7 (PMC9831989; doi:10.1038/s41598-023-27610-7)
Supplement: Supplementary file 1 — Supplementary Information. [file 41598_2023_27610_MOESM1_ESM.pdf]

Supplementary Material

Modulation of trigeminal neuropathic pain by optogenetic inhibition of posterior hypothalamus in CCI-ION rat

Jaisan Islam[1], Elina KC[1], Kyoung Ha So[2,6], Soochong Kim[3], Hyong Kyu Kim[4], Yoon Young Park[5], Young Seok Park[1,2,5*]

1. Department of Medical Neuroscience, College of Medicine, Chungbuk National University, Cheongju, Korea.
2. Institute for Stem cell & Regenerative Medicine (ISCRM), College of Veterinary Medicine, Chungbuk National University, Cheongju, Republic of Korea.
3. Department of Veterinary Medicine, College of Veterinary Medicine, Chungbuk National University, Cheongju, Korea.
4. Department of Medicine and Microbiology, College of Medicine, Chungbuk National University, Cheongju, Korea.
5. Department of Neurosurgery, Chungbuk National University Hospital, Cheongju, Korea.
6. Bio-MAX/N-Bio Institute, Institute of Bio-Engineering, Seoul National University, Seoul, Republic of Korea.

Supplementary Method: Chronic constriction injury of infraorbital nerve surgery. General anesthesia in animals were accomplished with an intraperitoneal (i.p.) injection of a mixture of 15 mg/kg Zoletil (Zoletil50®, Virbac Laboratories, Carros, France) and 9 mg/kg Rompun (Rompun®, Bayer, Seoul, South Korea) in saline. After that they were mounted onto the surgical field in a prone position. The skin above the eye was then shaved, and then the animals were placed in a stereotaxic frame. To prevent any damages associated with drying of the eyes, ophthalmic ointment was applied to the cornea. A skin incision was made along the curve of the frontal bone in the anterior-posterior direction, 2 mm above the left eye. Moving laterally, the fascia and muscle were gently separated from the bone using a periosteal elevator. The infraorbital nerve (ION) could be observed on the maxillary bone following retraction of the eye. Once revealing the ION, we prepared for ligature placement by gently freeing approximately 8 mm of the ION from the surrounding connective tissue. The ION was stretched slightly using a blunt needle with a curved head for ligature placement. The two ligatures were gently placed 3-4 mm apart, following which they were tightened until the ION was barely constricted. Finally, the incision above the eye was sutured with silk (3-0).

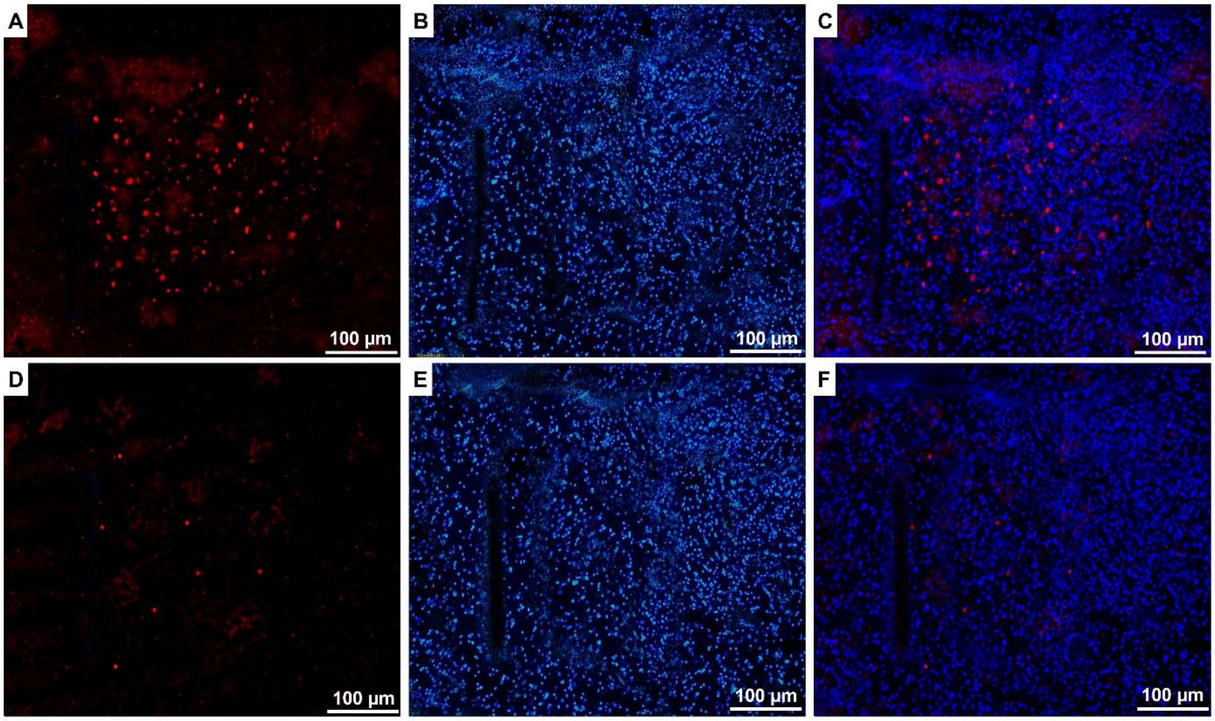

Supplementary Figure 1: Immunofluorescence results of c-Fos expression following CCI-ION surgery. (A-C) Increased c-Fos expression in the posterior hypothalamus of TNP animals. (A) expression of c-Fos-positive neurons, (B) DAPI, (C) Merged. **(D-F)** No or, very little c-Fos expression in the posterior hypothalamus of sham animals. (D) expression of c-Fos-positive neurons, (E) DAPI, (F) Merged. Scale bar = 100 µm.

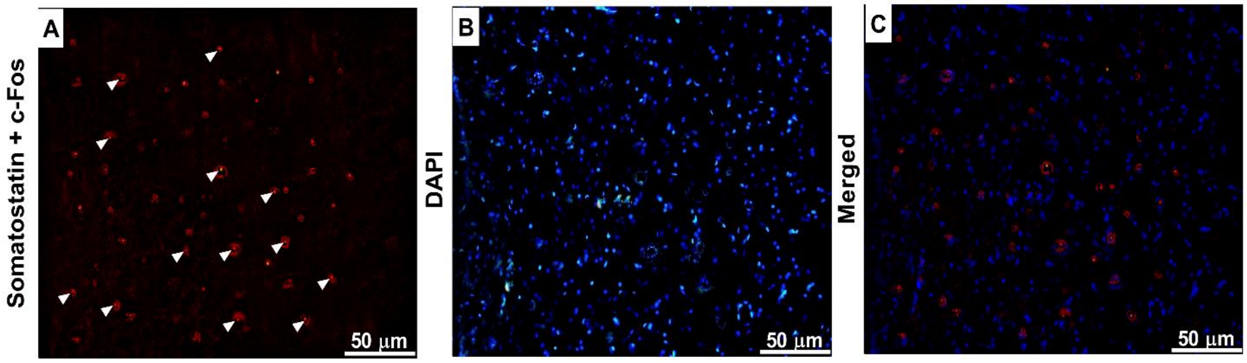

Supplementary Figure 2: Immunofluorescence results of somatostatin antibody. (A-C) Somatostatin neurons in the posterior hypothalamus. (A) colocalization of somatostatin and c-Fos (white arrowhead), (B) DAPI, (C) Merged. Scale bar = 50 µm.

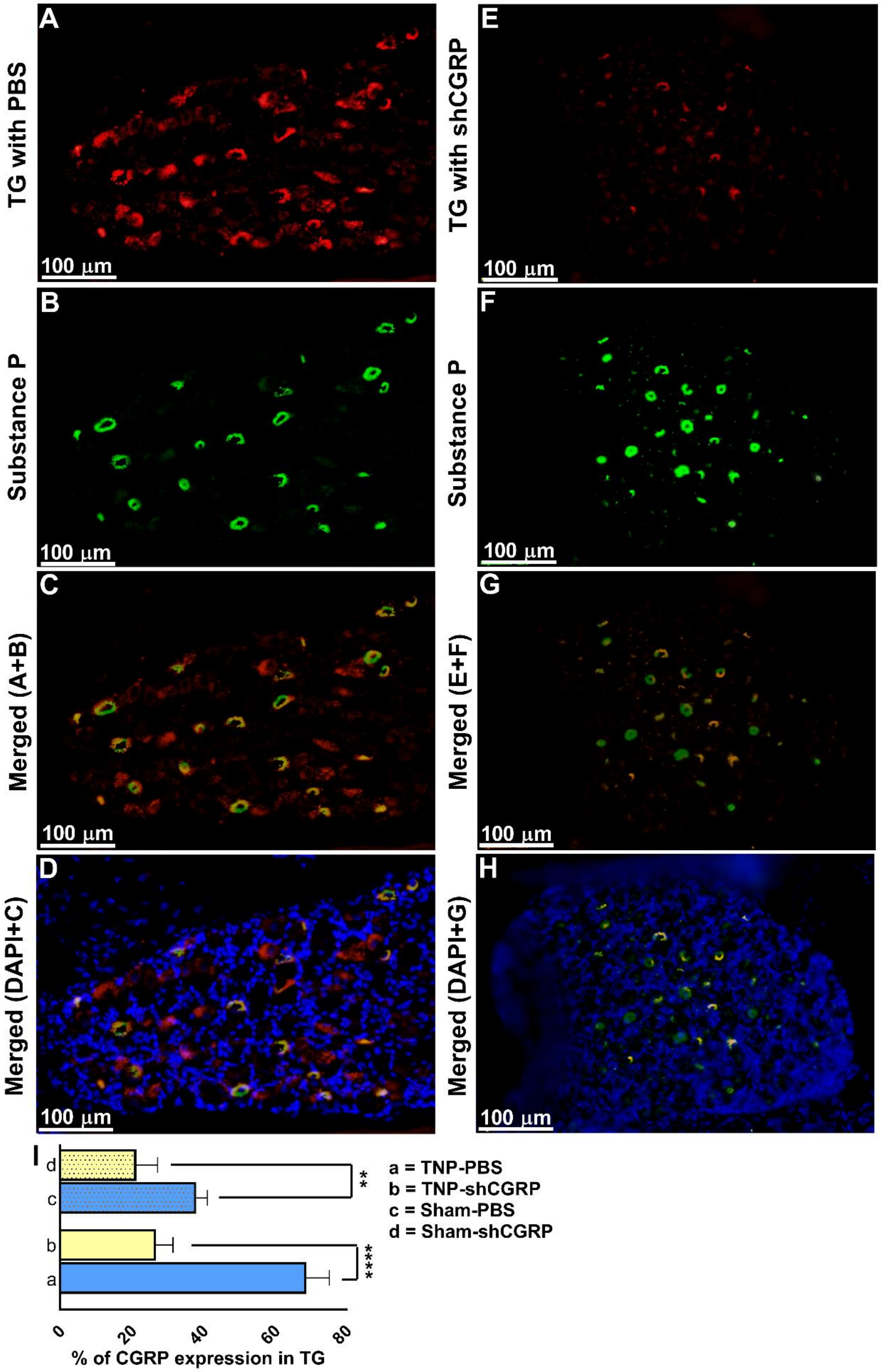

A
TG with PBS
100 μm
B
Substance P
100 μm
C
Merged (A+B)
100 μm
D
Merged (DAPI+C)
100 μm
E
TG with shCGRP
100 μm
F
Substance P
100 μm
G
Merged (E+F)
100 μm
H
Merged (DAPI+G)
100 μm
I
d
c
b
a
**
****
0
20
40
60
80
% of CGRP expression in TG
a = TNP-PBS
b = TNP-shCGRP
c = Sham-PBS
d = Sham-shCGRP

Supplementary Figure 3: CGRP expression in TG of PBS- and shCGRP-injected animal. (A-D) Presence of anti-CGRP antibody binding due to the active state of α-CGRP neurons within the trigeminal ganglion of PBS-injected animals. (E-H) shCGRP-injected trigeminal ganglion cells showing the absence of expression of CGRP neurons due to the inhibition of α-CGRP neurons within the trigeminal ganglion. (A, E) CGRP, (B, F) Substance P, (C, G) Merged (CGRP+Substance P), and (I, L) Merged (CGRP+Substance P+DAPI). Scale bar = 100 µm. (I) Quantification of the CGRP expression (expressed in percentage) in the TG of TNP-CGRP (n=8), TNP-PBS (n=8), Sham-CGRP (n=8) and Sham-PBS (n=8) group animals. Significant changes in presence of active CGRP were observed between shCGRP injected animals' TG and PBS injected animals' TG. **, $p < 0.01$; ****, $p < 0.0001$, significant differences determined using unpaired *t*-test. Data are displayed as means ± SD.

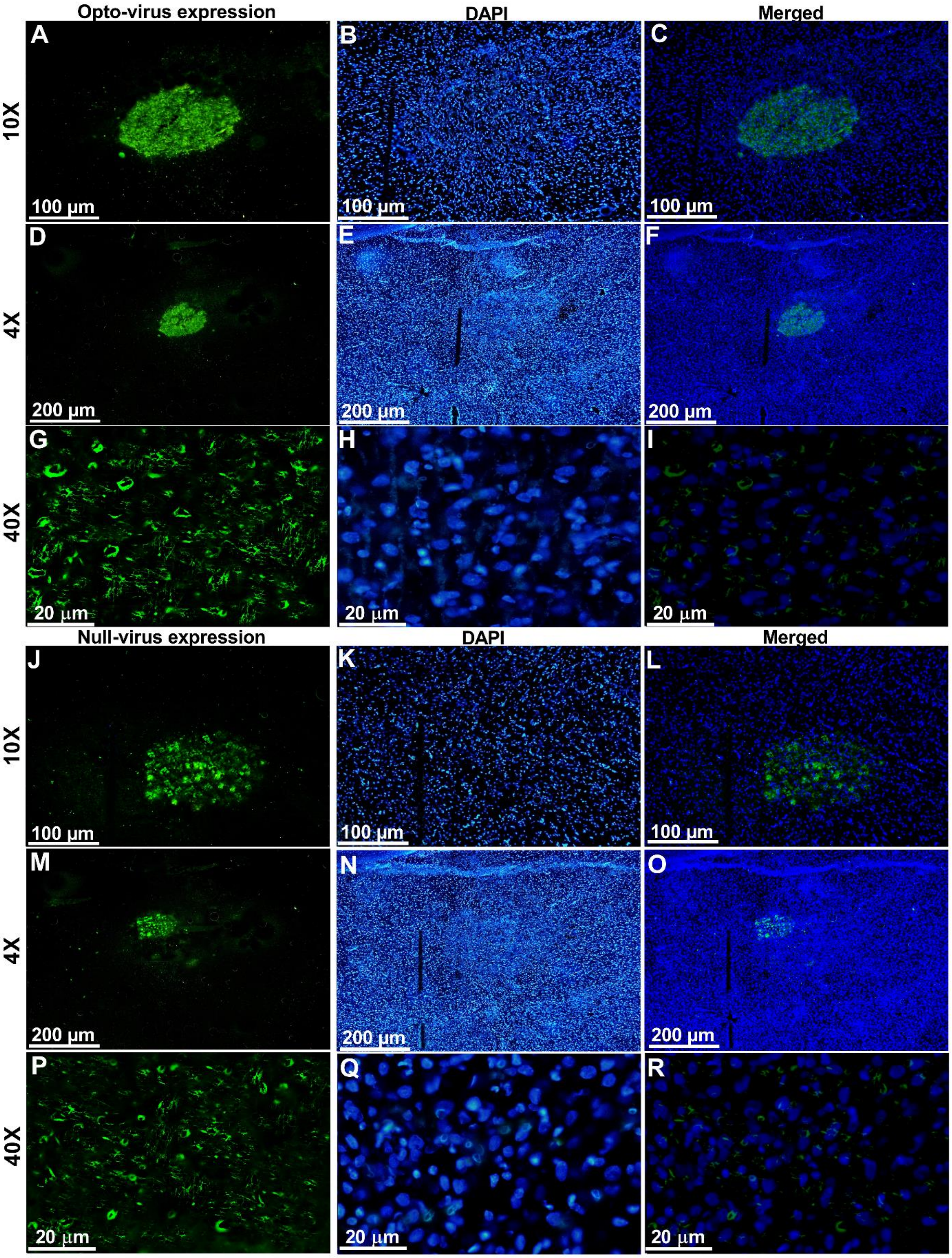

Opto-virus expression
DAPI
Merged
10X
4X
40X
A
B
C
D
E
F
G
H
I
100 µm
200 µm
20 µm
Null-virus expression
J
K
L
M
N
O
P
Q
R

Supplementary Figure 4: Immunofluorescence results of optogenetic and null virus expression at different magnifications. (A-C) Opto virus expression with 10x magnification. (D-F) Opto virus expression with 4x magnification. (G-I) Opto virus expression with 40x magnification. (J-L) Null virus expression with 10x magnification. (M-O) Null virus expression with 4x magnification. (P-R) Null virus expression with 40x magnification. (A-C, J-L) Scale bar = 100 µm. (D-F, M-O) Scale bar = 200 µm. (G-I, P-R) Scale bar = 20 µm.

Supplementary Table 1: Results of the open field test of the TNP, sham, and control groups after CCI-ION surgery.

Group	TNP (n=32)		Sham (n=32)		Control (n=4)	
	Baseline	4th week	Baseline	4th week	Baseline	4th week
Distance travelled	66 ± 8	43 ± 9 **	63 ± 11	64 ± 12	67 ± 12	68 ± 8
Rearing events	58 ± 8	33 ± 8 **	56 ± 11	58 ± 12	62 ± 12	63 ± 9
Activity time	393 ± 25	216 ± 20 ****	388 ± 23	383 ± 32	371 ± 30	396 ± 28
Exploration rate	283 ± 25	171 ± 13 ****	278 ± 19	276 ± 18	267 ± 21.5	289 ± 17
Grooming time	32 ± 5	44 ± 5 ***	32 ± 6	30 ± 7	30 ± 6	29 ± 5

TNP = trigeminal neuropathic pain animal. Values are displayed as the means ± standard deviation. **$p < 0.01$; ***$p < 0.001$; ****$p < 0.0001$ (repeated measures one-way ANOVA).

Supplementary Table 2: Results of the open field test of the TNP-Opto-CGRP, TNP-Opto-PBS, TNP-Null-CGRP, and TNP-null-PBS groups with and without optogenetic stimulation at week 7-9.

Group	TNP-Opto-shCGRP (n=8)		TNP-Opto-PBS (n=8)		TNP-Null-shCGRP (n=8)		TNP-Null-PBS (n=8)	
	Stim-OFF	Stim-ON	Stim-OFF	Stim-ON	Stim-OFF	Stim-ON	Stim-OFF	Stim-ON
Distance travelled (m)	32.16 ± 2.32	43.62 ± 2.87 *	31 ± 2.6	42.33 ± 2.34 *	31.86 ± 2.32	31.5 ± 2.4	30.38 ± 1.93	32.25 ± 1.30
Rearing events	27.75 ± 4.12	41.38 ± 3.20 **	29 ± 2.74	39 ± 3.21 *	27.16 ± 3.76	27.88 ± 3.72	31.13 ± 2.71	29.75 ± 2.22
Activity time (sec)	219.88 ± 9.24	249.88 ± 7.29 **	216.87 ± 8.74	238.86 ± 7.30 *	221.5 ± 8.20	217.66 ± 6.30	220.25 ± 8.02	220.88 ± 10.28
Exploration rate	166.5 ± 8.82	195.86 ± 12.01 **	164.38 ± 12.26	192.18 ± 10.56 *	159.66 ± 12.53	159.16 ± 10.1	164 ± 7.14	167.66 ± 8.38
Grooming time (sec)	41.38 ± 3.43	35 ± 2.65 *	44.66 ± 4.09	36.28 ± 3.16 *	44.13 ± 3.89	45.5 ± 3.81	46.5 ± 3.39	46.5 ± 2.35

Stim-ON = Stimulation-ON condition, Stim-OFF = Stimulation-OFF condition. * = statistical comparison between Stim-ON and Stim-OFF condition, Values are displayed as the means ± standard deviation. *$p < 0.05$; **$p < 0.01$ (Paired *t*-test).

Supplementary Table 3: Effect of CGRP-knockdown on behavioral responses of TNP animal.

Behavior test	TNP-Opto-shCGRP (n = 8) (Before stimulation)	TNP-Opto-shCGRP (n = 8) (After stimulation)	Statistical significance of improvement	TNP-Opto-PBS (n = 8) (Before stimulation)	TNP-Opto-PBS (n = 8) (after stimulation)	Statistical significance of improvement
Air-puff test	13.98 ± 1.75 psi	17.38 ± 1.84 psi	3.4 ± 0.6435 psi Unpaired *t*-test (t, df) = (6.284, 14) $P < 0.01$ **	13.11 ± 1.69 psi	16.08 ± 1.86 psi	2.97 ± 0.8885 psi Unpaired *t*-test (t, df) = (4.343, 14) $P < 0.05$ *
Cold hyperalgesia test	32.38 ± 2.14	26.29 ± 1.86	6.09 ±0.9002 Unpaired *t*-test (t, df) = (6.075, 14) $P < 0.01$ **	33.18 ± 2.05	28.03 ± 1.96	5.15 ± 0.8013 Unpaired *t*-test (t, df) = (4.206, 14) $P < 0.05$ *
Von Fray filament test	11.86 ± 1.69 g	14.079 ± 1.53 g	2.219 ± 0.8060 g Unpaired *t*-test (t, df) = (3.053, 14) $P < 0.05$ *	10.71 ± 1.51 g	13.88 ± 1.34 g	3.1 ± 0.1138 g Unpaired *t*-test (t, df) = (3.804, 14) $P < 0.05$ *
Distance traveled	32.16 ± 2.32 m	43.62 ± 2.87 m	11.46 ± 1.305 m Unpaired *t*-test (t, df) = (5.003, 14) $P < 0.05$ *	31 ± 2.6 m	42.33 ± 2.34 m	11.33 ± 1.237 m Unpaired *t*-test (t, df) = (4.397, 14) $P < 0.05$ *
Rearing events	27.75 ± 4.12	41.380 ± 3.20	13.63 ± 1.844 Unpaired *t*-test (t, df) = (7.003, 14) $P < 0.01$ **	29 ± 2.74	39 ± 3.21	10 ± 1.492 Unpaired *t*-test (t, df) = (5.8, 14) $P < 0.05$ *
Activity time	219.88 ± 9.24 s	249.88 ± 7.29 s	30 ± 4.161 s Unpaired *t*-test (t, df) = (7.210, 14) $P < 0.01$ **	216.87 ± 8.74 s	238.86 ± 7.3 s	21.99 ± 4.026 s Unpaired *t*-test (t, df) = (5.462, 14) $P < 0.05$ *
No. of explored area	166.5 ± 8.82	195.86 ± 12.01	29.36 ± 5.268 Unpaired *t*-test (t, df) = (5.573, 14) $P < 0.01$ **	164.38 ± 12.26	192.18 ± 10.56	27.80 ± 5.721 Unpaired *t*-test (t, df) = (4.859, 14) $P < 0.05$ *
Grooming time	41.38 ± 3.43 s	35 ± 2.65 s	6.380 ± 1.532 s Unpaired *t*-test (t, df) = (4.163, 14) $P < 0.05$ *	44.66 ± 4.09 s	36.28 ± 3.16 s	8.38 ± 1.83 s Unpaired *t*-test (t, df) = (4.296, 14) $P < 0.05$ *

TNP = trigeminal neuropathic pain animal. Values are displayed as the means ± standard deviation. *$p < 0.05$; **$p < 0.01$ (Unpaired *t*-test).
